# Recycling Potential of Plastic Resources from End-of-Life Passenger Vehicles in China

**DOI:** 10.3390/ijerph181910285

**Published:** 2021-09-29

**Authors:** Yang Li, Shiyu Huang, Yanhui Liu, Yiyi Ju

**Affiliations:** 1 School of Business Administration, Zhongnan University of Economics and Law, Wuhan 430073, China; liyang@zuel.edu.cn (Y.L.); huangshiyu6699@163.com (S.H.); liuyanhui7737@163.com (Y.L.); 2 Waseda Institute for Advanced Study, Waseda University, Tokyo 1698050, Japan; 3 Institute for Future Initiatives, The University of Tokyo, Tokyo 1130033, Japan

**Keywords:** plastic waste, end-of-life passenger vehicles, recycling, waste management, extended producer responsibility

## Abstract

A rapid increase in the number of end-of-life (EoL) passenger vehicles has led to a large amount of waste plastics in China. However, the scale and efficiency of recycling resources from EoL vehicles still restricts the sustainable and healthy development of the automotive industry. The current behavior of automotive/recycling industry entities, as well as the strategy of waste management policymakers, may depend on the potential of total recyclable resources. To reveal such recycling potential of various plastic materials in EoL passenger vehicles, we predicted total EoL passenger vehicles in China from 2021 to 2030 (used the Weibull distribution) considering passenger vehicle ownership (estimated by the Gompertz model), quantified the demand for new passenger vehicles (estimated using its non-linear relationship with income level and passenger vehicle ownership), and assessed the recyclable plastics by categories and by provinces. The results show that (i) the annual average recycled plastic resources from EoL vehicles would exceed 2400 thousand t in 2030, more than 2.5 times in 2021, showing a great recycling potential; (ii) the differences among the three scenarios are relatively small, indicating that no matter the saturation level of passenger vehicles in China would be high or low, a rapid increase of recyclable plastic resources can be expected from 2021 to 2030; (iii) at the provincial level, a considerable gap between the potential of recycling plastic from EoL passenger vehicles and the regional processing capacity. Given such great potential and regional differences, the recycling policies should be applied in stages and consider the development level and recovery pressure in each region.

## 1. Introduction

Lightweight, strong, durable, and low-cost plastics are used in a wide range of manufactured products worldwide [1]. In 2018, global plastic output was close to 360 million t, of which China’s plastic output was close to 110 million t, accounting for about 30% of the global total [2]. In recent years, with the advent of “lightweight” development within the automobile industry, the proportion of plastics used in vehicles has also gradually increased. In addition, the increasing end-of-life (EoL) passenger vehicles have led to the production of a large amount of waste plastics. Determining how to manage these waste plastics more efficiently has become one of the most important problems worldwide [3]. 

Waste plastics require space for landfills, which has caused great economic loss due to increasingly scarce urban land resources. In addition, many landfilled waste plastics cause severe damage to landforms, vegetation, and the natural landscape [4]. Some waste plastics can even release harmful and toxic gases, causing severe air pollution and health problems [5,6,7]. Waste plastics landfilled arbitrarily or without proper anti-leakage technology can severely pollute the soil at disposal sites [8,9] and disrupt the food chain [10]. If waste plastics are discharged directly into surface water systems (oceans, lakes, or rivers), large amounts of floating debris will reduce water area and threaten the survival of aquatic plants [11,12,13,14]. Waste plastic is the main factor contributing to marine pollution [15,16]. Every year, 515 million t of plastic are discharged into the sea, induced by direct pollution from beaches and marine waste, and pollution from freshwater ecosystems, atmospheric transportation, and fishing and shipping activities, among many other sources [17,18]. Plastic waste in the marine environment kills animals and plants, threatens food security, and seriously endangers human health [5,19].

Plastic recycling is necessary for minimizing pressure on natural resources [20]. Recycling automotive plastic, especially high-value recycling, can not only protect the environment but also save and effectively utilize nonrenewable resources to their greatest extent, significantly contributing to a circular economy [21,22]. However, China’s potential for plastic waste recycling is still largely undeveloped. In addition to issuing a plastic ban, China urgently needs to strengthen its management of waste plastic recycling [23], which depends mainly on the type of plastic material [24]. 

The potential to recycle renewable resources from ELVs (end-of-life vehicles) has aroused the interest of many scholars. In China, ELV means a motor vehicle that has reached the national end-of-life standard or, although it has not reached the national end-of-life standard, the engine or chassis is severely damaged and has been inspected and does not meet the national technical conditions for the operation of motor vehicles or does not meet the national motor vehicle emission standards for pollutants. The automotive industry uses a large amount of metal resources (including steel and aluminum) and non-metallic resources (including plastics and rubber). The production and sales of passenger vehicles are occupying a large share (over 80%) of the industry, so it is of great significance to study the resource recycling potential of end-of-life passenger vehicles [25]. Regarding research methods, material flow analysis (MFA) [26,27] and life cycle assessment (LCA) [28,29] have been used to analyze resource consumption and environmental impact. However, most studies analyzing recyclable resources from EoL passenger vehicles have been based on production and sales [30,31]. The potential for recyclable resources mainly depends on the number of ELVs, which is further related to many factors such as vehicle ownership and income. It is necessary to quantify the key factors into available data for accurate, comprehensive, and comparable research. Due to the high recycling value of metal materials, previous studies have mainly focused on metal resources [32,33,34], and analyses of plastic recycling are rare. Among them, Jiang et al. [35] utilized information from the automotive industry to conduct historical calculations and scenario analyses based on the dynamic material flow model to research plastic flow and the stock of passenger vehicles in China from 1950 to 2050. Jin et al. [36] studied Japan’s ELV recycling industry at the time and their prospects for plastic recycling. However, plastics contain many polymers, increasing the difficulty of their effective recovery. The recycling method for a given plastic material depends on the type of polymer, the product design, and even whether the producer is responsible for collecting/recycling the product vs. another party [37], and it is closely related to the recycling capacities of the enterprises in each region. As plastics are the most used in passenger vehicles, second only to steel, analyzing the regional potential and capacity for recycling the various polymers in plastics can provide basic data, predictions regarding policy, and treatment suggestions for coping with the mountains of plastic waste.

Therefore, in order to effectively manage waste plastics, it is very important to predict the recycling potential of the various plastic materials in EoL passenger vehicles. In this paper, we use the Weibull distribution to predict the number of EoL passenger vehicles in China from 2021 to 2030, according to passenger vehicle ownership (estimated using the Gompertz model) and demand for new passenger vehicles (estimated using its non-linear relationship with income level and passenger vehicle ownership). Furthermore, we assess which plastics from EoL passenger vehicles are recyclable and analyze the differences in the plastic recovery potential and capacity among all provinces in order to provide important references for the secondary utilization of plastic materials as well as to determine the extent of the development of extended producer responsibility (EPR) in China.

## 2. Materials and Methods

### 2.1. Estimation of Passenger Vehicle Ownership per 1000 People

In China, many factors will affect the future passenger vehicle demand and passenger vehicle ownership, and ultimately affect the generation of EoL passenger vehicles. Therefore, improving and updating the existing modeling methodology is the best approach [38]. There are many factors affecting the ownership of passenger vehicles, which can be summarized as policy factors (such as automobile popularization policy and transportation policy), social factors (such as population density, road network density and accessibility of public transport facilities), economic factors (such as per capita GDP, income level, vehicle price and use cost) [39]. However, at the national level of China, Per capita GDP explains most of the changes in vehicle ownership [40].

As for the prediction of vehicle ownership, according to the historical data of vehicle ownership in developed countries, the development trend of vehicle ownership per 1000 people is similar to the growth of per capita GDP, showing a “slow- urgent- slow” S-shaped [39,41]. The commonly used S-shaped curves include Richards function, logistic function and Gompertz function [42]. Among the nonlinear functions, the Richards function is a growth curve function with four parameters, which has strong adaptability to data fitting. However, the estimation of its parameters is complex. Which method to choose depends on the availability of data. The data accumulation period of China’s automobile industry is short and the data availability is poor, some mature methods are difficult to obtain the necessary data. Therefore, we can only use simpler methods that are easy to obtain data [38]. In the prediction of vehicle ownership, the special forms of Richards function: Logistic function and Gompertz function are more widely used [41,43]. Tanner took the lead in using a logistic function to analyze the per capita vehicle ownership rate in Britain as early as 1958 [44]. Compared with the logistic function and Richards function, the Gompertz function can better describe the development of vehicle ownership [38,39]. The S-shape is not necessarily central symmetric in the Gompertz function, which allows the later or earlier stages to witness a relatively more rapid development of vehicle ownership rate. Such features well fit the theoretical development pattern of vehicle ownership rate [41,45].

**Figure 1:**
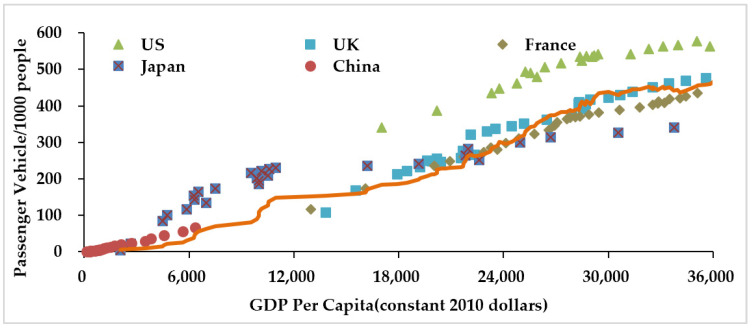
Passenger vehicle ownership per 1000 people in developed countries and China. The data of GDP and population from World Bank Open Data; passenger vehicle ownership from World Motor Vehicle Statistics (2016).

We selected four typical developed countries, US, UK, France, Japan, as well as China, to reveal the relationship between vehicle ownership (specifically, passenger vehicle) per 1000 people and GDP per capita shown in Figure 1. It may also reveal the development pattern of passenger vehicle ownership.

In the past 50 years, the ownership of passenger vehicles in developed countries has increased significantly along with income increases. Although the four countries varied in the ownership rates with the same income level of their residents, they share the same increasing trend of the ownership rates in response to the GDP per capita, specifically, with a slow-rapid-slow development pattern shown as an S-shape of moving average line in Figure 1. In the first stage, the ownership rate grows slowly as the income level is low and passenger vehicles are not affordable for most households. In the second stage, the ownership rate grows rapidly in response to the increasing income level. In the third stage, along with the popularization of passenger vehicles, the vehicle ownership rate becomes close to its saturation level and grows slowly again. In the case of China, researches [38,46] also proved that similar to the mentioned developed countries, the development of passenger vehicle ownership in China also fits the positive correlation with income level and a slow-rapid-slow pattern with a saturation level in the later stage. However, since the automobile society in China is still in the beginning stage, such a trend cannot be observed as clear as in developed countries [39].

In this study, the Gompertz function was adopted to model the vehicle ownership rate in China at a national level from 2021 to 2030. Therefore, the estimated passenger vehicle ownership per 1000 people in year t can be formulated as

(1) s(t)=K exp(α exp(βg(t)))

where variable g(t) represents the GDP per capita, revealing the impact of a long-term economic trend; parameter K represents the saturation of passenger vehicle ownership; α and β are parameters. Parameter K is introduced under three scenarios that consider the saturation of passenger vehicle ownership in different developed countries. The determination of the ultimate saturation level is essential to the estimation of passenger vehicle ownership. When the national vehicle market reaches saturation, the growth in the passenger vehicle ownership rate may stay stable at around zero. In this paper, we defined a national saturation is reached when the growth rate of its passenger vehicle ownership per 1000 people over four straight years fell into the range from −1.5% to 1.5%. Furthermore, we set up three scenarios to determine possible levels of saturation. Parameters α and β, describing the shape of s(t), could be calculated based on existing data from before 2020.

The GDP per capita g(t) was estimated using the exponential relationship fitting China’s observed data. According to the National Bureau of Statistics of China, China’s 2018 GDP reached 90 trillion yuan (13.6 trillion USD), and the GDP per capita reached roughly USD 10,000. Moreover, researchers from the China Association for Labor Studies anticipate that, by the end of 2050, the population of middle-income earners in China will reach 900 million, 60 percent of the country’s predicted total population [47]. If China’s GDP growth rate stays at approximately 6% for the next 10 years, its GDP per capita may reach the high-income standard of USD 12,736 defined by the World Bank in 2015. Therefore, an exponential relationship was adopted because the growth rates of income levels in developing countries are believed to increase along with the processes of urbanization and industrialization, especially in China. The GDP per capita g(t) can be formulated as

(2) $$g\left(t\right)=\delta e^{\mu t}$$

where variable t ranges from 1 to 71, representing the years 1960 to 2030; parameters δ and μ describe the shape of g(t) and can be estimated based on existing data from 1960 to 2020.

### 2.2. Estimation of Passenger Vehicle Demand

The demand for new passenger vehicles is estimated using its non-linear relationship with the modeled passenger vehicle ownership per 1000 people and the income level. Both axes may affect the estimated value of the demand. TableCurve 3D software (TableCurve 3D software, San Jose, CA, USA) was utilized to illustrate the shape of the demand for new passenger vehicles. It can be formulated as

(3) $$P\left(s,g\right)=a+b{\left(\ln s\right)}^{2}+c\ln g$$

where P represents the demand for new passenger vehicles, s represents the passenger vehicle ownership per 1000 people, and g represents the GDP per capita.

Parameters a, b, and c describe the shape of P. Parameters a, b, and c can be calculated based on existing data regarding the demand for new passenger vehicles, which is defined as the sum of the consumption and the net import of new passenger vehicles.

### 2.3. Estimation of EoL Passenger Vehicles

In order to predict the number of future EoL passenger vehicles, it is necessary to fit the characteristics of vehicle scrapping. In general, due to differences in vehicle uses, road conditions, and mechanical properties, vehicles entering the market in the same year are scrapped year by year, and there is a scrapping peak. The Weibull distribution can fit this feature well; therefore, it has been widely applied in the lifespan estimation of vehicles [48,49].

In this paper, the Weibull distribution function is used to estimate the number of EoL passenger vehicles. As the passenger vehicles registered in year t do not retire in the same year, the position parameter is set to zero. Therefore, the accumulated retirement rate of EoL passenger vehicles is formulated as in Equation (4):

(4) $$F\left(t\right)=1-exp\left[-{\left(t/\eta \right)}^{m}\right]$$

where F(t) represents the accumulated retirement rate in year t, which follows the Weibull distribution; η is the scale parameter; and m is the shape parameter (m>0,η>0).

The vehicle ownership is formulated as in Equation (5):

(5) $$\hat{S}\left(t\right)=\overset{k}{\underset{n=1}{\sum }}P\left(t,n\right)*\left(1-F\left(t\right)\right)$$

where $\hat{S}\left(t\right)$ represents the estimation of passenger vehicle ownership in year t, n represents the vehicle age, *k* represents the maximum year for passenger vehicles to be retired, and P(t,n) is the passenger vehicle demand in year (t−n). Therefore, the scale parameter, η, and the shape parameter, m, can be calculated using the least-squares method.

Thus, the number of EoL passenger vehicles can be formulated as in Equation (6):

(6) $$\hat{W}\left(t\right)=\overset{k}{\underset{n=1}{\sum }}P\left(t,n\right)*F\left(t,n\right)-\overset{k}{\underset{n=1}{\sum }}P\left(t-1,n\right)*F\left(t-1,n\right)$$

where $\hat{W}\left(t\right)$ represents the total number of EoL passenger vehicles in year t and F(t,n) is the accumulated retirement rate with a vehicle age of n years in year t.

### 2.4. Estimation of Recyclable Plastics from ELVs

There are many types of plastics in passenger vehicles. In this paper, the total weight of recyclable plastics from EoL passenger vehicles is estimated using their various plastic contents. The total weight of recyclable plastics from EoL passenger vehicles in year *t*, $\hat{Q}\left(t\right),$ can be formulated as

(7) $$\hat{Q}\left(t\right)=\overset{h}{\underset{j=1}{\sum }}\hat{W}\left(t\right)clrw_{j}$$

where c denotes the average weight of passenger vehicles, l denotes the share of plastic in passenger vehicles, r denotes the plastic recycling efficiency of EoL passenger vehicles, $w_{j}$ denotes the share of the *j*th type of plastic contents, and *h* denotes the number of categories of plastics.

### 2.5. Data Sources

In this paper, the vehicle ownership rate per 1000 people is from the China Automotive Industry Yearbook (2017)’s [50] World Motor Vehicle Statistics (2016) [51]. The indicators of saturation of vehicle ownership (in the US, the UK, France, and Japan) and the Chinese GDP per capita are from the World Bank Open Data [52]. The demand for new passenger vehicles is from the China Automotive Industry Yearbook [50]. The average weights and structures of plastics from passenger vehicles are from the literature [35,53]. The provincial ownership of passenger vehicles comes from the China Statistical Yearbook [54]. The use of plastics in vehicles has nearly tripled in the last 30 years [55]. With increasing national requirements regarding the recycling rates of EoL passenger vehicles and the development of high-performance engineering plastics, the proportion of plastics used in vehicles will be even higher in the future. The application of plastics in passenger vehicles has evolved from their use in ordinary decorative parts to their use in important structural and functional parts. Materials have also expanded from ordinary plastics to stronger and more impact-resistant composites or plastic alloys. The main materials used are polypropylene (PP), polyethylene (PE), polyvinyl chloride (PVC), ABS, polyurethane (PU), polyamide (PA), polycarbonate (PC), polymethyl methacrylate (PMMA), polyformaldehyde (POM), etc. [56]. From the perspective of the current variety and proportion of automotive plastics, PP has become the most used variety of automotive plastics due to its excellent molding properties and cost performance [35].

Most automotive plastics are of high quality and provide good performance, and plastic parts of scrapped passenger vehicles still have a high reuse value [57]. Regarding the recycling rate of passenger vehicle materials, the European Union issued a new “End-of-Life Vehicle Recycling Directive” in 2006 [58]. Newly listed vehicles in the EU market must ensure that the material recycling rate accounts for more than 85% of the weight. The “Automobile Product Recycling Technology Policy” issued by China in 2006 requires that, from 2017 onwards, the recycling rate of materials for all domestic and imported M and N vehicles should be no less than 85% [59]. Therefore, this study uses 85% as the plastic recycling efficiency of EoL passenger vehicles.

## 3. Results

### 3.1. Passenger Vehicle Ownership per 1000 People in China

The saturation level of passenger vehicle ownership rate (K) estimated by this paper is: 516 per 1000 people in US, 473 per 1000 people in UK, 433 per 1000 people in France, and 438 per 1000 people in Japan. Compared to the saturation level of vehicle ownership estimated by existed literature: 800 per 1000 people in US (including cars and light-duty trucks) [60], 450–600 per 1000 people in European countries [61], and 440 per 1000 people in Japan [62], the results of this paper can be regarded as reliable. Based on the estimated passenger vehicle ownership rate in mentioned developed countries, this paper set three scenarios for the passenger vehicle ownership rate in China, however, considering the fact that China’s automobile market has not yet entered the saturation stage, as well as that the domestic economic development is unbalanced. The three scenarios are: (i) 430 per 1000 people under BAU scenario (Business as Usual), (ii) 510 per 1000 people under high-saturation-level scenario, (iii) 350 per 1000 people under low-saturation-level scenario.

The GDP per capita of China (g) from 2019 to 2030 estimated by this paper is:

(8) $$g\left(t\right)=0.0713exp\left(0.0734t\right)\left(R^{2}=0.9818\right)$$

where variable t ranges from 1 to 71, representing years 1960 to 2030. If the annual GDP growth rate of China stays at 6% from 2031 onward, the passenger vehicle ownership rate will reach saturation in 2044 under the BAU scenario, in 2042 under the low-saturation scenario, and in 2046 under the high-saturation scenario; if the annual GDP growth rate of China stays at 5% from 2031 onward, the passenger vehicle ownership rate will reach saturation in 2047 under the BAU scenario, in 2044 under the low-saturation scenario, and in 2049 under the high-saturation scenario. It can be observed that, in the years in which passenger vehicle ownership rates reach saturation, the high- and low-saturation scenarios do not vary to a great extent, which indicates that, in the case of China, preference of passenger vehicle and income level do not greatly affect the timing of reaching saturation. The results also support the urgency and importance of estimating the potential production of EoL passenger vehicles and of improving their recycling. As shown in Table 1, in 2030, passenger vehicle ownership will reach saturation at 377 per 1000 people under the BAU scenario, at 321 per 1000 people under the low-saturation scenario, and at 427 per 1000 people under the high-saturation scenario. When compared with the saturation of vehicle ownership estimated by the existing literature (25.0% of households owning at least one vehicle in 2030 [45], 390 million vehicles total in 2030 [41], and 250–290 trillion passenger vehicles in 2020 [63]), the results of this paper can be regarded as reliable.

### 3.2. Demand for New Passenger Vehicles

The demand for new passenger vehicles (*P*) from 2021 to 2030 estimated by this paper is as follows:

(9) $$P=199\;{\left(\ln\left(s\right)\right)}^{2}-1106\;\ln\left(g\right)-267,\left(R²=0.9949\right)$$

shown in Figure 2.

**Figure 2:**
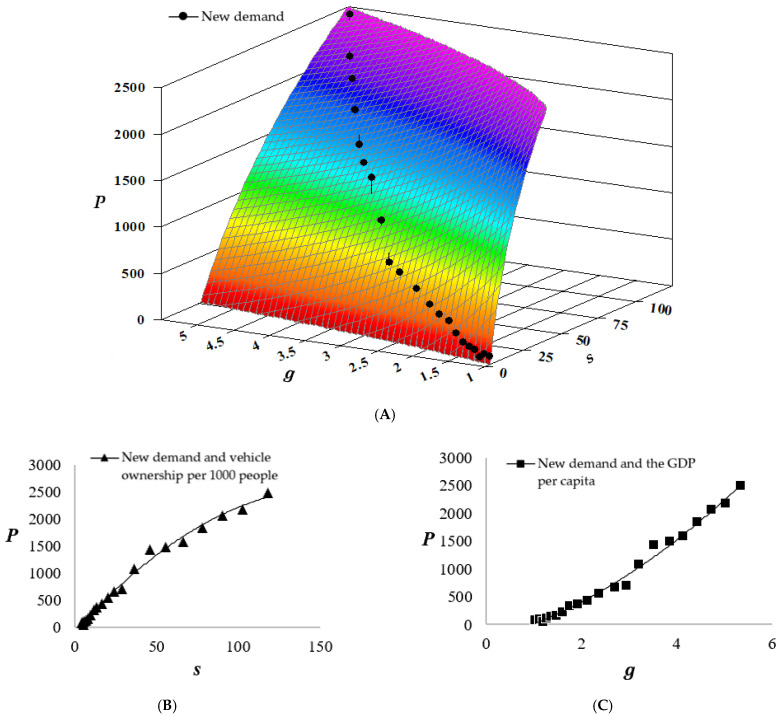
Estimation of the demand for new passenger vehicles (**A**). The relationship between *P* and *s* (**B**). The relationship between *P* and g (**C**). The unit of P (estimated demand for new passenger vehicles) is $10^{4}$ vehicles; the unit of s (estimated passenger vehicle ownership per 1000 people) is vehicles/1000 people; and the unit of g (GDP per capita at constant prices) is $10^{4}$ yuan.

As the demand for new passenger vehicles was estimated using its non-linear relationship with both ownership and income level, both axes affected the estimated value and had the same positive correlation but in opposite convexities. P increased dramatically when s was relatively small (<50) and slowly when g was relatively small (<2). The trendlines (second-order polynomial) in Figure 2B,C also show the opposite convexity of the two relationships.

The demand for new passenger vehicles is compared with the volume of EoL passenger vehicles in Section 3.3.

### 3.3. Generation of EoL Passenger Vehicles

The parameters describing the shape of the survival rate of passenger vehicles (the scale parameter, η, and the shape parameter, m) in this paper are m=3.257 and η=14.286. The results show that, for vehicles of the same age, few of them retired in the first 7 years, the retirement rate reaches a higher level from year 10 to year 15 and peaked at year 13 with an annual retirement rate of 9% and survival rate of 48%, and the survival rate reaches approximately 0% at year 24. The results regarding the survival rate and the retirement rate reflect China’s current situation and developmental pattern in terms of preference of vehicles and ongoing EoL vehicle policies. In 1986, a compulsory retirement standard was implemented in China and was officially announced as the “automobile scrapping standard” in 1997. According to the standard, the mandatory scrapping standard for passenger vehicles is 10 years (or a service distance over 100 thousand kilometers). With the rapid development of the automobile industry in China, the restriction was extended to 15 years in 2000. In the latest “standard for compulsory scrapping of motor vehicles”, announced in 2013, the mandatory retirement age for small- and medium-size non-operating passenger vehicles was canceled (instead, the service distance limitation was set at 600 thousand kilometers) [64]. If passenger vehicles travel an average of 20 thousand kilometers per year, their service time can be 24–30 years.

**Figure 3:**
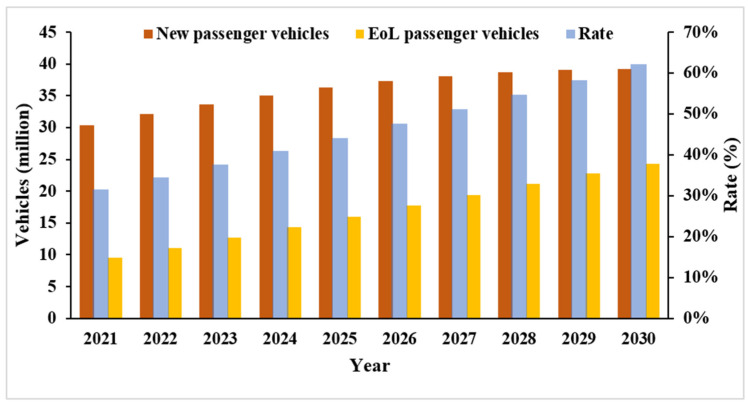
Estimations of new passenger vehicle demand and EoL passenger vehicles from 2021 to 2030 (under the BAU scenario).

The new passenger vehicle demand (P) and the total number of EoL passenger vehicles ($\hat{W}$) from 2021 to 2030 estimated by this paper are shown in Figure 3.

In 2021, under the BAU scenario, it is estimated that the number of EoL passenger vehicles will reach 9.56 million, accounting for 31% of the demand for new passenger vehicles (30.35 million). By 2030, EoL passenger vehicles will reach 39.19 million, accounting for 62% of the demand for new vehicles (24.33 million). It can be observed that the gap between the two will narrow in the next decade, namely because the effective recycling of ELV resources can greatly alleviate the pressure on resources and the environment. Therefore, many countries have put forward recycling requirements for producers to implement EPR systems in the automotive industry.

### 3.4. Recyclable Plastics from EoL Passenger Vehicles in China

Assuming that all EoL passenger vehicles enter the formal recycling channel and that the material recovery rate of EoL passenger vehicles is 85%, the total recyclable plastics from EoL passenger vehicles (${\hat{Q}}_{j}$) from 2021 to 2030 are shown in Table 2.

It can be observed that, under the three scenarios, the average annual amount of plastic recovered from EoL vehicles will exceed 2400 thousand t in 2030, more than 2.5 times that in 2021, showing great recycling potential.

**Figure 4:**
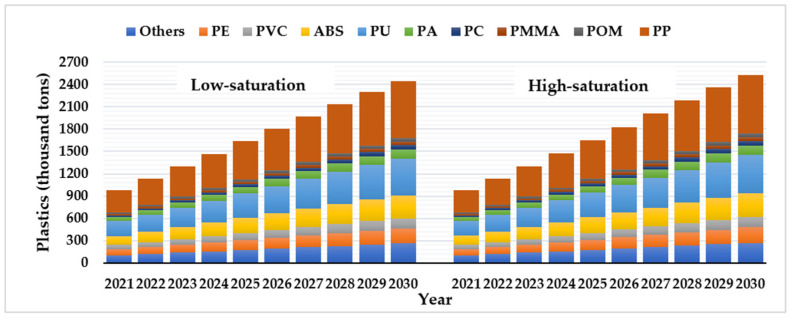
Various types of recyclable plastics from EoL passenger vehicles from 2021 to 2030.

Plastic materials can be recycled in many ways, and their waste management depends largely on the type of plastic [24]. Various types of recyclable plastics from EoL passenger vehicles in China from 2021 to 2030 are shown in Figure 4 within two different scenarios.

In terms of the composition of passenger vehicle plastic materials, the consumption of PP is the highest, and its recovery could reach 761.92–788.44 thousand t in 2030 (Figure 4). According to the average price of recyclable plastic in sub-regional markets in China [65], the potential economic value of PP could reach 3.90–4.03 billion yuan (603.4–623.6 million USD), which shows great recycling value. In 2030, the resource recovery potential for PU, ABS, PE, and PVC, which account for a large proportion of recyclable plastics, will reach 491.47–508.57 million t, 307.90–318.62 thousand t, 202.04–209.08 thousand t, and 138.16–142.97 thousand t, respectively. The differences in total recyclable plastics from EoL passenger vehicles among the three scenarios are relatively small (+1% and −2% compared with the BAU in 2030). Therefore, no matter whether the saturation of passenger vehicles in China is high or low, a rapid increase in the population of waste plastics can be expected from 2021 to 2030.

**Figure 5:**
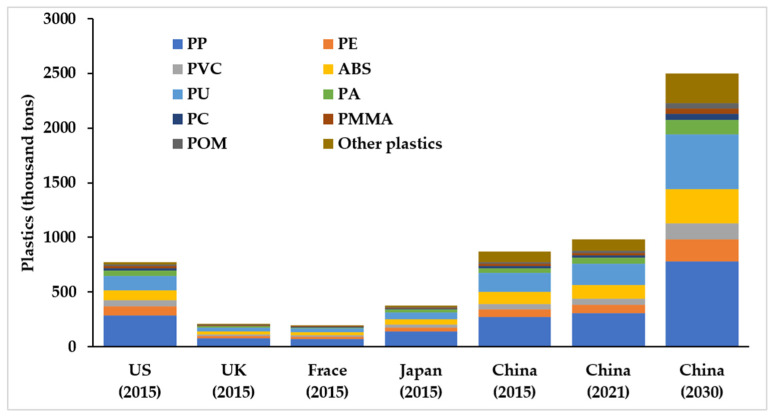
The potential of recyclable plastics from EoL passenger vehicles in multiple countries.

In developed countries, theoretically scrap quantity of vehicles accounts for 6–8% of the vehicle ownership volume [66]. Therefore, we estimate the theoretical recyclable plastics from EoL passenger vehicles in developed countries [67] and make an international comparative analysis, with the same recovery setting of this study. The results are shown in Figure 5. Under the BAU scenario, in 2015, the theoretical recyclable plastics of passenger vehicles in China (867.45 thousand t) has slightly exceeded that in the US (775.47 thousand t), about 2.3 times that in Japan. By 2030, the theoretical recyclable plastics from EoL passenger vehicles (under the BAU scenario) will be 2.9 times that in 2015, showing great recycling potential.

## 4. Present Situation and Problems Regarding the Recycling of Plastics from EoL Passenger Vehicles

### 4.1. Current Situation and Problems

**Figure 6:**
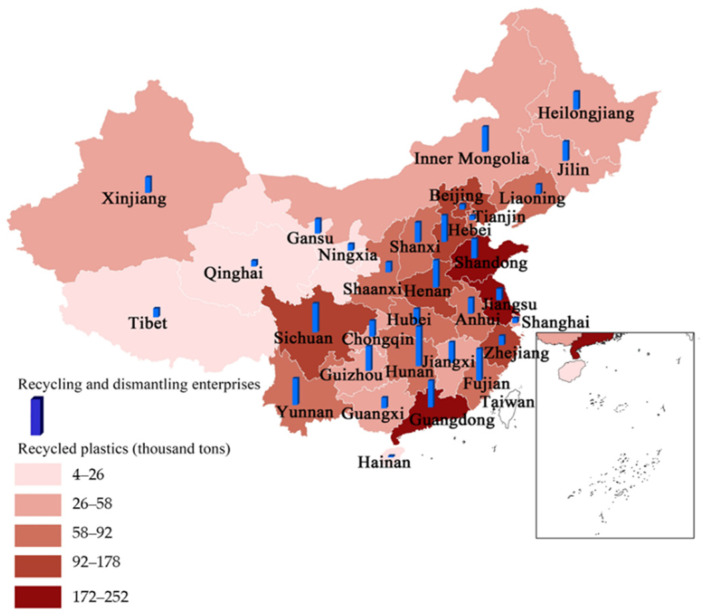
The spatial distribution of EoL passenger vehicle plastic recycling potential in China’s provinces in 2030 (under the BAU scenario).

In order to further explore the current situation and the problems involved with plastic recycling, based on the above prediction data, we estimated the provincial distribution of recyclable plastics from EoL passenger vehicles in China. As the scrap volume of passenger vehicles in each province is closely related to local ownership [66], we normalized the regional ownership of passenger vehicles for a sum of 1 to obtain the weight index of the scrap volume. Finally, we distributed the scrap volume according to weight to estimate the provincial distribution of plastic recycling potential. The estimated spatial distribution of automobile plastic recycling potential in China’s provinces in 2030 is shown in Figure 6.

At the provincial level, recyclable plastics from EoL passenger vehicles vary greatly among regions. Under the BAU scenario, the three provinces with the highest plastic recycling potential are Guangdong (252 thousand t), Shandong (226 thousand t), and Jiangsu (194 thousand t), and the lowest three provinces are Tibet (4 thousand t), Qinghai (10 thousand t), and Ningxia (12 thousand t). These results are related to their respective economic development levels and populations. The areas with the most recyclable plastic resources are the most developed areas in China. However, these areas have high land prices, high labor costs, and insufficient landfills, so the cost and pressure of handling a large number of EoL passenger vehicles are even greater. The actual recovery of plastics is not only related to recovery potential but also related to the specific recovery system and recovery capacity. Due to the total amount of control implemented by the government over ELV recycling and dismantling enterprises (only 755 formal enterprises in 2019), the number of large-scale recycling enterprises is insufficient, and some enterprises are still operating at a low level [66,68]. Therefore, there is a huge gap between the potential of recyclable plastic and regional processing capacity. At present, the ELV recycling system is not perfect, and the plastic recycling policy is not detailed enough, resulting in a low level of reuse for high-value-added waste plastics. Therefore, China has not yet established an effective waste plastic recycling system [69].

### 4.2. Promoting Recycling of Plastics from EoL Passenger Vehicles Based on EPR

Since 2017, China has vigorously implemented the EPR system into the automotive industry [70], aiming to build a more effective resource recycling system for ELVs. EPR is defined as “An environmental policy in which a producer’s responsibility for a product is extended to the post-consumer stage of a product’s life cycle” [71] and has been adopted in many countries to make producers responsible for managing their products until the post-consumer stage.

In practice, due to differences in the social, economic, and technical factors, compared with developed countries, China’s EPR regulations for plastic management are not specific enough [72,73]. Therefore, on the one hand, China can learn from the experiences of developed countries, refine the classification standards of plastic products, improve the enterprise environmental information disclosure system, promote the implementation of the EPR system through legal requirements and economic incentives, and establish an EPR performance evaluation and supervision system. On the other hand, due to China’s unbalanced regional development and the different pressures regarding waste plastic recycling in different regions, more effective legal norms should be formulated according to regional characteristics, and the EPR system should be improved in stages.

## 5. Conclusions

With the introduction of lightweight vehicles and the development of high-performance engineering plastics, many metal parts on vehicles are being replaced by plastic parts. The amount of high-performance engineering plastics has become one of the key indicators of the development level of the modern automobile industry [74]. There are various types of automotive plastics used in passenger vehicles. After various types of plastics are processed into parts, they are assembled and manufactured in a vehicle factory, and they enter society along with the use of passenger vehicles. Next, they follow the law of life distribution and become waste plastics as vehicle parts are replaced or vehicles are scrapped. This study estimates the recyclable plastic potential of EoL passenger vehicles based on a forecast of their numbers in China from 2021 to 2030.

The results show that:

(i). the average annual amount of plastic recovered from EoL vehicles would exceed 2400 thousand t in 2030, more than 2.5 times that recovered in 2021, showing great recycling potential. Under the BAU scenario, 777.26 thousand t of PP (polypropylene), 501.36 thousand t of PU (polyurethane), 314.10 thousand t of ABS, and 206.11 thousand t of PE (polyethylene) will be recycled.
(ii). In terms of weight, the differences in waste plastics from EoL passenger vehicles among the three scenarios are relatively small. No matter the saturation of passenger vehicles in China is high or low, a rapid increase in the population of recyclable plastic resources can be expected from 2021 to 2030. Considering such great potential of plastic resources, policy instruments such as EPR may provide solutions for more sustainable development.
(iii). However, at the provincial level, there is a huge gap between the potential of recyclable plastic from EoL passenger vehicles and the regional processing capacity. For developing countries such as China, based on the experience of developed countries, the EPR system can be improved in stages. The application of it should consider the development level and recovery pressure in each region.

Many factors will affect the prediction of future demand and passenger vehicles ownership, and ultimately affect the projection of EoL passenger vehicle generation in this case of China. Two main aspects can be further explored as our next steps. First, traffic policy adjustments (such as purchase support/restriction policies, traffic control policies, and mandatory scrapping policies) may bring some uncertainties to the prediction of passenger vehicle ownership. Also, factors such as access to other public transport alternatives will affect the saturation level of vehicle demand. Secondly, this study may benefit more from a detailed investigation into the current prediction of overall passenger vehicles, by categorizing them as private light passenger vehicles, commercial light passenger vehicles, etc.

## Figures and Tables

**Table 1:** Estimation of passenger vehicle ownership per 1000 people from 2021 to 2030.

Year	BAU	Low-Saturation Scenario	High-Saturation Scenario
2021	182	174	188
2022	206	194	215
2023	230	214	242
2024	254	234	271
2025	279	252	300
2026	302	269	329
2027	324	285	356
2028	344	299	382
2029	361	311	406
2030	377	321	427
Parameter/Feature	BAU	Low-saturation scenario	High-saturation scenario
α	−6.3115	−6.3708	−6.3101
β	−0.2955	−0.3276	−0.0273
$R^{2}$	0.9994	0.9989	0.9995

**Table 2:** Estimation of total recyclable plastics from EoL passenger vehicles from 2021 to 2030 (thousand t).

Year	BAU	Low Saturation	High Saturation
2021	981.07	980.04	982.10
2022	1136.03	1133.98	1137.06
2023	1300.23	1297.15	1302.28
2024	1470.58	1464.42	1473.66
2025	1645.04	1635.80	1651.20
2026	1820.52	1807.18	1829.76
2027	1994.98	1975.48	2008.32
2028	2166.36	2139.68	2185.86
2029	2334.66	2297.72	2361.34
2030	2496.80	2447.55	2532.72
